# Wood Composites and Their Polymer Binders

**DOI:** 10.3390/polym12051115

**Published:** 2020-05-13

**Authors:** Antonio Pizzi, Antonios N. Papadopoulos, Franco Policardi

**Affiliations:** 1 LERMAB-ENSTIB, University of Lorraine, 88000 Epinal, France; 2 Department of Forestry and Natural Environment, International Hellenic University, 66100 Drama, Greece; antpap@for.ihu.gr; 3 Faculty of Electrical Engineering, University of Ljubljana, Tržaška cesta 25, SI-1000 Ljubljana, Slovenia; franc.policardi@fe.uni-lj.si

**Keywords:** wood composites, wood composite binders, synthetic wood adhesives, biosourced wood adhesives, environment-friendly, new approaches

## Abstract

This review presents first, rather succinctly, what are the important points to look out for when preparing good wood composites, the main types of wood composites manufactured industrially, and the mainly oil-derived wood composite adhesives and binders that dominate and have been dominating this industry. Also briefly described are the most characteristic biosourced, renewable-derived adhesives that are actively researched as substitutes. For all these adhesives, synthetic and biosourced, the reviews expose the considerable progresses which have occurred relatively recently, with a host of new approaches and ideas having been proposed and tested, some even implemented, but with even many more already appearing on the horizon.

## 1. Introduction

Wood composites is a growing field of products that are increasingly present for a variety of applications, with an undiminished upward trend now for very many decades. One must first define what is strictly intended as a wood composite as there are a host of different products that, while they could be classified as wood composites, are really on the margin of what is defined as such in the jargon of the wood profession. In general, two main and distinct groups of wood composites exist, namely, strictly speaking, just wood panel composites and the rest, this latter being glulam, fingerjoints, etc., with parallam and scrimber really belonging in the margin of both classes. In general, also, a wood composite is a composite in which the wood is in a markedly dominant proportion. This would exclude wood–plastic composites where the proportion of the plastic is equal, or almost equal, to the proportion of the wood present, and they are excluded from this review.

It must be clearly pointed out that one cannot speak about wood composites without speaking in depth of the polymer binders and adhesives used to hold them together. The history of wood composites themselves is inextricably intertwined with the history and the development of the polymer binders that hold them together and their manufacture. In fact, not only has there been continuous development of new or improved binders that has allowed the development of wood composites but, as presented later in this review, it is the continual renewal, new discovery, and upgrading of such binders that has allowed and allows progress in wood composites. It is for this reason that this review is divided into three parts. The first describes the main wood composites existing today, the second the synthetic adhesives that have dominated and still dominate this field, and the third the variety of new adhesives, either synthetic or biosourced, or renewables, which is the real area of intellectual ferment and exploration today. This is defined with the clear understanding that for any adhesives or composites where in-depth reviews already exist, the reader will be referred to these. This approach is adopted as the field is really very vast, as befits a wood binders industry that represents in excess of 65%, by volume, of all the adhesives produced in the world, all applications included.

It must be also pointed out that the technique to prepare a good wood composite is the mix of two very different technologies, namely the use of a good adhesive, hence the capacity of formulating, engineering, and preparing one, and the technology of manufacture of the composite, with this being particularly true in the case of wood panels. Thus, to obtain a good/acceptable wood composite is a 50/50 balance between these two technologies. This must be clearly kept in mind because one can prepare really awful wood panel composites even when using an excellent adhesive if the composite assembly technology is faulty and, conversely, one can still prepare an acceptably good composite by playing with its manufacturing technology, even if the adhesive is rather mediocre, if not outright poor. Thus, the mastering of the two technologies is essential. The manufacturing techniques of wood composites are mechanical and will not be treated in this review, which is polymers-oriented; nonetheless, the most essential examples will be presented in brief. In-depth, detailed reviews of the manufacturing technology of pressing and on how to master this to obtain a good wood panel composite already exist, and the readers are referred to these extensive specialized reviews [1].

## 2. Wood, Wood Plasticization and Wood Panels

Wood is a natural composite made of approximately 60−65% carbohydrate fibers (approx. two-thirds cellulose and one-third hemicelluloses), 25−30% of a random polyphenolic branched polymer, lignin, functioning as a fiber binder, and 10% of residues, extractives, or cellular waste infiltrates (oleoresins, tannins, starches, some inorganic salts, etc.) coating its porous cellular surfaces. Wood panel manufacturing is based on the densification of a particle mat and its consolidation in a hot press. At a molecular level, wood cells are deformed above their elastic domain and plastic deformation or damage occurs [1]. Both mechanisms result in an irreversible deformation. As wood viscoelastic properties depend on internal mat conditions, when the environment is such that the viscous component becomes very important, flow takes place, and this leads to a true plastic deformation. If conditions do not allow or inhibit molecular movement, damage will appear. Thus, mechanisms that occur in wood densification and are regarded as a manifestation of plasticity would, under different circumstances, be considered as damage. Referring to wood composites, most of the final board properties depend on how wood cells buckle [2].

**Figure 1:**
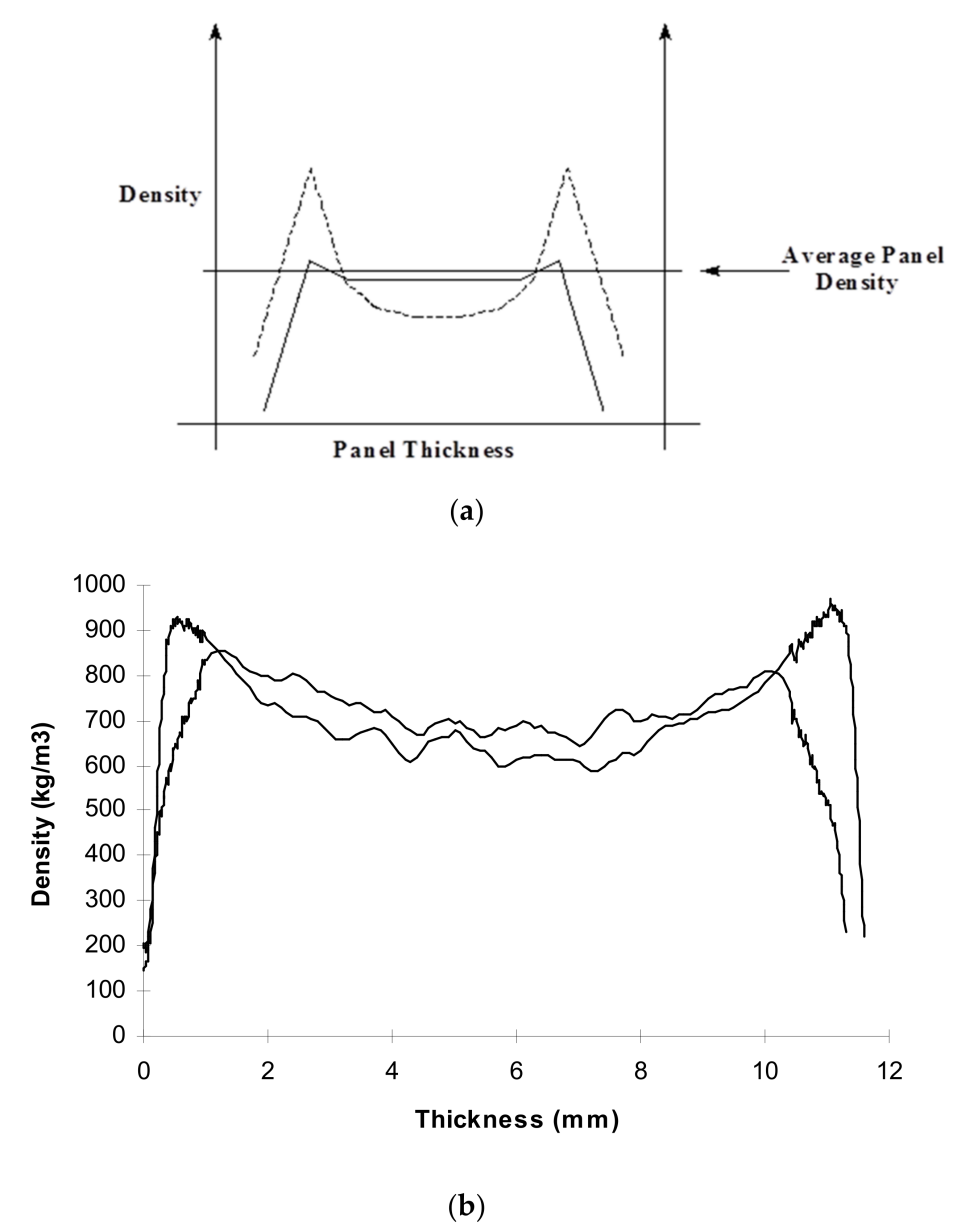
(**a**) Schematic, exaggerated representation of two different panel density profiles along the panel thickness leading to very different board properties; (**b**) an example of two real, different density profiles of two different wood particleboards [1].

Among the many factors influencing the performance of the composite, the relative moisture content of the panel’s surfaces and core is the most important as one can easily play on it to improve a composite by its pressing technology, even if the adhesive is not a great one. This concept leads to the importance of the density profile across the thickness of a wood composite panel. The density profile as a function of panel thickness is an important measure and a forecasting tool of the likely characteristics of a panel. At parity of overall panel density, the shape of the density profile determines which are the characteristics that the panel will have. The different panel density profiles are shown in Figure 1, in an exaggerated manner, for example purposes.

First of all, the density of the surfaces is practically always higher than the density of the core of the board, if the board is well made. The low density of the outermost surfaces of the raw panel is always eliminated in industrial panels just by sanding, so that the surfaces can present the highest density possible, hence, as hard as possible. Panels which have relatively higher surface densities and lower core densities at parity of total panel density have better bending strength and better screw-holding capability. Conversely, panels which have relatively lower surface densities in relation to the core density have better internal bond strength (tensile strength perpendicular to the panel plane) and better durability. Thus, what type of strength characteristics and, hence, which density profile it should be manufactured with often depends on the application which is intended for the board. Many parameters influence the compression ratio in the press and, hence, the density profile of the panel. In the introduction of this chapter, it has been stated that one can still make good panels when using a relatively poor adhesive. This can be explained on the basis of the density profile. Internal bond strength depends almost exclusively on how good the adhesive is, and on how high is the density of the panel layer of lower density. When the adhesive is poor, it is then sufficient to use pressing conditions of the panel that increase compression of the core to bring it to a higher density value, and lowers the density of the surfaces without changing the panel overall density. A flatter profile is then obtained, such as the flatter profile in Figure 1, where the weakest layer has a higher density, hence giving a much higher internal bond strength, this being the real measure of how good the panel and bonding are. This can be achieved quite readily by just changing, for example, the relative percentage moisture content (MC%) of surfaces and core before pressing. Thus, changing the surface to core MC% of 14%:10%, which is quite common, to just 13%:11% or 12%:10% will considerably densify the board core and upgrade the internal bond strength of the board [1]. In this respect, other parameters also have strong influence on this effect, such as the rate of press closure, maximum pressure, and others [1]. In industrial practice, however, a multipurpose board satisfying national standards is often produced unless the client has pre-specified the type of characteristics they want.

## 3. Types of Wood Composites

Many types of wood panels, for a variety of applications, are manufactured today. Their definitions, in brief, are as follows, but the reader is referred to far more detailed and in-depth reviews on the technology of manufacturing of these products [1,3,4].

**Particleboard**: A flat hot-pressed wood composite panel composed of randomly oriented wood chips bonded by hot-pressing by using thermosetting adhesive resins, mainly urea–formaldehyde (UF), melamine–urea–formaldehyde (MUF), phenolic resins (PF and TF), and isocyanates (pMDI). The board is generally composed of three distinct layers, the surface layers being composed of finer wood chips than the coarser core layers. Some processes yield a continuously chip-size-graded board along the surface/core/surface thickness. The panel generally has a density of 650−700 kg/m^3^ and the average amount of resin solids in the board core section is of between 6% and 12% on dry wood (although lower and much higher percentages are also sometimes used). Panels of the same type but composed of wood chips of greater length and greater width but similar thickness are called also flakeboard and waferboard.

**Oriented Strand Board (OSB):** A flat hot-pressed three-layers wood composite panel composed of oriented wood wafers bonded by hot-pressing by using thermosetting adhesive resins. The very thin wafers (length and width are very much bigger than in particleboard and of the order of 100 mm × 20 mm, respectively) are oriented in the same direction within the same layer and at 90° of each other in adjacent layers yielding a particularly strong panel very suitable for structural applications. It is the modern competitor of plywood but at a much lower price. The lower surface area of the wafers, in relation to other types of panel, yields panels that need to be bonded with only 4−5% adhesive solids on dry wood. OSB is, today, the main substitute panel for the rather more expensive plywood, but presenting the same advantages. It is a panel for structural use.

**Medium Density Fiberboard (MDF):** a flat hot-pressed composite panel composed of wood fibers obtained by thermomechanical wood pulping and traditionally bonded with an adhesive to a density of around 750–800 kg/m^3^. MDFs of much lower densities are also known. It is a panel mainly bonded with urea–formaldehyde resins, and used for furniture and interior use. Its production has experienced a considerable growth.

**Hardboard (high density fiberboard):** A flat-pressed wood composite panel composed of randomly oriented wood fibers obtained by thermomechanical wood pulping and traditionally bonded without any adhesive by hot-pressing simply by the very high density (900–1100 kg/m^3^) and the high-temperature-induced flow of the lignin component of the fibers. Panels containing a small amount of adhesives (2–3% adhesive solids on dry fiber), generally PF resins, are often produced today to upgrade the properties of the panel.

**Plywood:** A flat hot-pressed multilayer wood panel composed of oriented wood veneers bonded by hot-pressing by using thermosetting adhesive resins. The veneer wood grains are oriented at 90° of each other in adjacent layers, yielding a particularly strong panel. As a consequence, this is the panel with the best strength/weight ratio but is rather expensive in relation to the equally strong OSB.

**Laminated Veneer Lumber (LVL):** A flat-pressed multilayer wood panel similar to plywood composed of oriented wood veneers but differently from plywood oriented all in the same direction in all the layers and bonded by hot-pressing by using thermosetting adhesive resins.

**Laminated beams (glulam), parallam, scrimber, and fingerjoints**: A flat-pressed multilayer wood beam with thick wood planks constituting the layers, used for structural exterior applications and bonded with PRF (phenol–resorcinol–formaldehyde) cold-setting resins, or MUF cold-setting resins, or even with certain types of polyurethanes (PURs), especially single-component PURs. The individual wood planks are bonded to the necessary length to compose the beam by fingerjoints bonded with one of the same three adhesives above.

Parallam and scrimber are similar products. Parallam is a beam made by a continuous manufacturing process composed of bigger size wood needles (very elongated wood particles) reassembled with a structural exterior grade adhesive, the favorite adhesive when heat curing isocyanates (pMDI) and PRFs when cold-curing. Scrimber was instead conceived by the CSIRO in Australia with the idea of “scrimming”, thus breaking down the structure of the wood by crushing it only as far as necessary and producing bundles of interconnected but still aligned strands to allow it to be formed by coating them with an adhesive into a desired end-product, rather than destroying the natural alignment of wood fibers and realigning them as more conventional processes do.

A recent review of the type of panels used in European industry is also available, and the reader is referred to this [4].

## 4. Current Wood Composite Adhesives

The most current big volume wood composite adhesives are briefly listed as follows. Synthetic adhesives do still dominate this market. What is already on the market will be described later for biobased adhesives and for totally new approaches in synthetic adhesives. The reader is however referred to far more in-depth reviews for each of them.

### 4.1. Urea–formaldehyde Adhesives

**Scheme 1:**
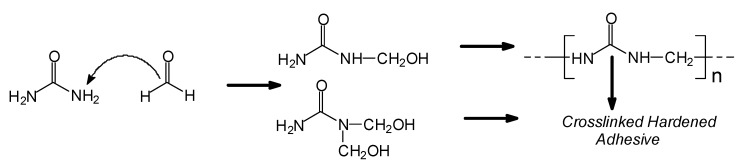
Basis reaction of urea and formaldehyde to prepare UF resin.

Urea–formaldehyde (UF) adhesives are, by far, the adhesive dominating the wood panel composites market for the preparation of interior grade composites used for furniture and a wide variety of other applications. An approximate volume of 11 million tons/year of these adhesives for wood are used worldwide. They are obtained by reaction of urea and formaldehyde according to the simplified Scheme 1:

Their technology has considerably progressed during the last few decades under the pressure of ever more stringent formaldehyde emission regulations. Notwithstanding their drawbacks, such as lack of resistance to exterior weather conditions and emission of formaldehyde, they are however very difficult to substitute due to their relatively low cost, their excellent adhesive performance, and their ease of handling. The reader is referred to more specific, in depth and detailed reviews on their chemistry and conditions of applications [5,6,7,8].

### 4.2. Melamine-Formaldehyde and Melamine–Urea–Formaldehyde Adhesives

**Scheme 2:**
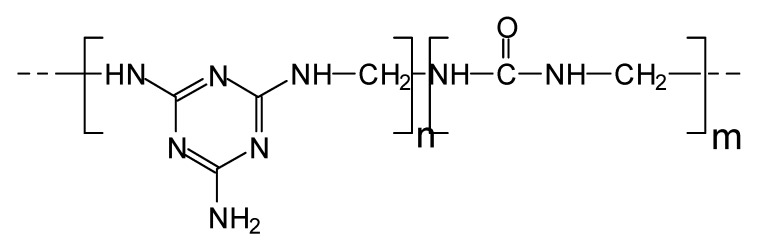
Schematic representation of a MUF resin.

Melamine is an expensive chemical, thus, today, these resins are traded as melamine–urea–formaldehyde (MUF) resins of equal performance as the older pure melamine–formaldehyde (MF) resins. They are of two types: (i) mUF, thus UF resins with just between 2–5% melamine that are nothing else than upgraded interior grade UF resins, this being also one method of decreasing a UF’s formaldehyde emission.; (ii) exterior- and semi-exterior-grade MUF adhesives containing between 30% to 40% of melamine. Their schematic representation can be shown in Scheme 2.

Traditionally, a few decades ago, these resins were considered as semi-exterior-grade, but the progress in their technology has been so considerable that they can compete well in performance with the more classical exterior-grade phenol–formaldehyde (PF) adhesives. Here, too, the reader is referred to more in-depth reviews [7,8,9].

### 4.3. Phenol–Formaldehyde Adhesives

**Scheme 3:**
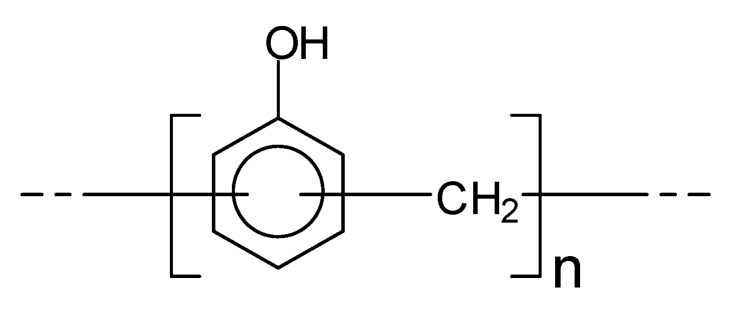
Schematic representation of a PF resin.

Phenol–formaldehyde (PF) adhesives are, by volume, the second most important wood composite adhesive, with up to 3 million tons/year being used worldwide. Their schematic structure can be represented as in Scheme 3

They are fully exterior-grade adhesives to bind truly weather-resistant wood composites and extensively used for particleboard, OSB, and marine plywood. They dominate where panels are used for housing, but have traditionally had the defect of having slower curing at higher temperature than for MUF adhesives. The progress has been considerable also in these resins, with PF adhesives now pressing as fast as MUF resins. Here, too, the reader is referred to more in-depth reviews [8,10,11].

### 4.4. Phenol-Resorcinol-Formaldehyde (PRF) Adhesives

**Scheme 4:**
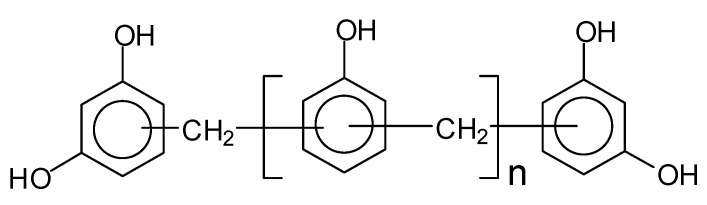
Schematic representation of a PRF resin.

Differently from the first three classes of wood composite adhesives presented above, PRF adhesives are cold-setting adhesives. They can be schematically represented in Scheme 4

They are expensive, due to the high cost of resorcinol that gives them their characteristic to cure at ambient temperature. They are thus used for glulam, finger-jointing, and similar products, and for ambient temperature-made LVL. They are binders for fully exterior-grade, weather-resistant composites. Due to their high cost and the particular products they are used to bind, their volume is relatively low, around 30 thousand tons/year, but they are high-value resins. Here, too, the reader is referred to more in-depth reviews [8,10,12].

### 4.5. Polymeric Isocyanates

**Scheme 5:**
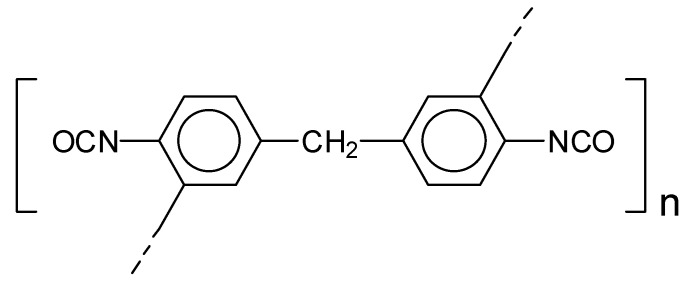
Schematic representation of pMDI.

The concern about the formaldehyde emission vapor levels from UF adhesives has brought isocyanate adhesives to the fore, where formaldehyde emission does not occur as no formaldehyde is added. For wood composites only polymeric 4,4′-diphenyl methane diisocyanate (pMDI) is the isocyanate used, which is a liquid of 100% solids. It can be schematically represented as (Scheme 5)

pMDI is an excellent adhesive and can be used in markedly smaller proportions than UF, MUF, and PF adhesives to bind wood composites. It is, though, relatively more expensive than the other three major formaldehyde-based adhesives, this somewhat counterbalancing the advantage of it being needed in lower proportions. Even this adhesive has more recently been under some pressure from stringent environmental regulations due to its vapor and relative toxicity. A use where it has a particular advantage is in mixing, in smaller proportions, with any of the three formaldehyde-based adhesives to upgrade their performance. Here, too, the reader is referred to more in-depth reviews [5,8,13].

### 4.6. One-Component Polyurethanes (PURs)

**Scheme 6:**
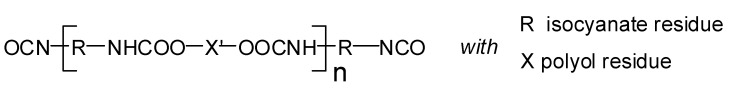
Schematic representation of monocomponent PUR.

One-component PURs are the main competitor of PRF adhesives for the same types of applications. Their structure can be schematically represented as (Scheme 6)

Their advantage is their considerably better ease of handling as no mixing with hardeners or catalysts are needed. Their main drawback is in the structure itself, as they are prone to yielding bonded joints subject to both creep and temperature-dependent creep. However, some of them have been formulated to partially overcome this drawback. They are relatively expensive, but this is not a drawback as their competitor, the PRF adhesive, is also expensive. Here, too, the reader is referred to more in-depth reviews [14].

## 5. New, Biobased, Renewable and Synthetic Wood Polymeric Adhesives

### 5.1. Biobased Wood Composite Adhesives

There are several biobased adhesives based on renewable natural materials that are at the forefront of new developments. Some of these are already industrial, sometime for many years, such as tannin adhesives and some soy adhesives, while others are on the way of industrialization, and many others are, as yet, at the experimental stage.

First of all, it is necessary to define what is meant by biobased wood adhesives or adhesives from renewable, natural, non-oil-derived raw materials. This is necessary because in its broadest meaning, the term might be considered to include urea–formaldehyde resins, urea being a non-oil-derived raw material. This, of course, is not the case. The term “biobased adhesive” has come to be used in a very well specified and narrow sense to only include those materials of natural, non-mineral origin which can be used as such or after small modifications to reproduce the behavior and performance of synthetic resins. Thus, only a limited number of materials can be currently included, at a stretch, in the narrowest sense of this definition. These are tannins, lignin, carbohydrates, unsaturated oils, proteins and protein hydrolysates, dissolved wood, and wood welding by self-adhesion.

Regarding tannin adhesives, tannins extracted from bark or wood of trees are traditionally used for leather, and these adhesives have been in industrial use since the early 1970s in a few countries of the southern hemisphere. There are a number of detailed reviews on the use of tannins for wood adhesives. The reader is referred to these detailed studies [8,15]. However, here, existing technologies and industrial use of tannin wood adhesives are presented.

As extensive studies already exist, regarding this application of tannin, only a few of the main achievements of tannin-based adhesives for wood products will be highlighted: (1) The development, optimization, and industrialization of non-fortified but chemically modified thermosetting tannins for particleboard, other particle products, and plywood [16,17,18]; (2) The technology for rapidly pressing tannin adhesives for particleboard, which is also industrial [19]; (3) The development and industrialization of tannin–urea–formaldehyde adhesives for plywood and, in particular, as impregnators for corrugated board starch binders [20,21]; (4) The development and industrialization of cold-setting tannin–resorcinol–formaldehyde adhesives for glulam and finger-jointing [22]; (5) The large-scale development and industrialization of quick-setting “honeymoon” separate application cold-setting adhesives for tannin-bonded glulam and fingerjoints [23,24,25]; (6) The development and industrialization of zinc salts to accelerate the hardening of non-fortified tannin adhesives for plywood [5,26,27,28]; (7) Successful formulation, development, and industrialization of pine bark tannin adhesives for particleboards and for glulam and finger-jointing in Chile [18,29]; (8) The development of isocyanate/tannin copolymers as difficult-to-bond hardwood adhesives and for plywood and other applications [30,31]; (9) The development of very low formaldehyde tannin adhesives for particleboards and other wood panels; (10) The development of the use of hardeners other than formaldehyde for thermosetting tannin adhesives [5,32,33,34]; (11) The discovery and development of self-condensation of tannin for adhesives [35,36,37,38,39,40,41,42].

All industrialized technologies today are based on paraformaldehyde or hexamethylene tetramine (hexamine) [42,43,44,45]. The latter is much more user- and environmentally friendly.

As regards new approaches in tannin-based wood adhesives, a number of experimental improvements have been studied, dictated by the new environment in which wood adhesives must operate. First of all, the relative scarcity of tannins produced in the world, compared to the tonnage of synthetic adhesives used in the panel industry, has led to a great deal of research on the extension of the tannin resource in order to have larger tonnage. As the potential material for tannin extraction shows that millions of tons of this material can be extracted each year worldwide, some companies have started to build additional extraction plants. This movement is still relatively small, but is ongoing. The second approach, to extend the tannin with another abundant and natural material, has led to the preparation of adhesives based on in situ copolymers of tannins and lignin [46] or copolymers of tannin and protein or soy flour [47], the use of tannin–furfuryl alcohol adhesive formulations, furfuryl alcohol being also a biobased material [48].

The second new constraint is the demand of most companies to eliminate formaldehyde emissions from tannin adhesive. This quest has taken two approaches: (i) total elimination of formaldehyde by substituting it with aldehydes that are less or nontoxic and nonvolatile [33,49], such as glyoxal, glutaraldehyde, or vanillin, the latter giving a fully biobased tannin adhesive and, more recently also, fully biobased carbohydrate extracts from very diffuse African trees that yield, on hot-pressing, both hydroxymethyl furfural and hydroxymethyl furfuryl alcohol as hardeners [34]; (2) the use of non-aldehyde hardeners such as tris(hydroxymethyl)nitromethane [50] and tris(hydroxymethyl)aminomethane [51] or even by combination with furfuryl alcohol, the latter functioning both as a hardener and a contributor to a tannin/furan copolymer [48,52]; (3) the use of hexamine with the formation of –CH_2_–NH–CH_2_– bridges between the tannin molecules, where the secondary amine is capable of absorbing any emission of formaldehyde from the heating of the wood itself or any other emission of formaldehyde to produce truly zero-formaldehyde emission panels [42,43,44,45]; (4) lastly, the hardening of the tannins by self-condensation without the addition of a hardener, self-condensation catalyzed by the wood substrate itself in the case of fast-acting procyanidin tannins, such as pine bark tannins, and for slower tannins, by addition of silica or silicate or other accelerators [8,35,36,37,38,39,40,41] allowing the preparation of wood particleboard of indoor quality.

A very new approach, giving very encouraging results, was obtained from the research on sodium periodate-specific oxidation of carbohydrates pursued in the ambit of the research on soy flour adhesives (see later) [53,54]. In this approach, periodate oxidation of the monomeric and oligomeric carbohydrates present in tannin extract and of glucose added to it caused cleavage of the monomeric and polymeric carbohydrates forming a variety of nontoxic and nonvolatile monomeric and oligomeric aldehydes. These reacted with the tannin polyphenolic part of the tannin extract to give good bonding results for particleboards [55].

**Lignin adhesives**: Extensive reviews on a number of proposed technologies of formulation and application do exist, and the reader is referred to these in earnest [56,57,58,59,60,61,62,63,64,65]. Lignin is the most abundant natural polymer after cellulose. Its polyphenolic nature has always generated interest for preparing wood adhesives. None of the many adhesive systems based on pure lignin resins, hence, without synthetic resin addition, have succeeded commercially at an industrial level. Some were tried industrially, but for one reason or another, too long a pressing time, high corrosiveness for the equipment etc., they did not meet with commercial success. Still notable among these is the Nimz system based on the networking of lignin in presence of hydrogen peroxide [8,56,66] and the Shen system based on the self-coagulation and crosslinking of lignin by a strong mineral acid in the presence of some aluminum salt catalysts [5,8,56]. Of interest in the MDF field is also the system of adding laccase enzyme-activated lignin to the fibers or activating the lignin, in situ, in the fibers, also by enzyme treatment with the addition of 1% polymeric isocyanate [66,67]. The more traditional approaches are the use of methylolated lignin or lignosulphonates in PF resins [68] or even the preparation of copolymerized lignin–phenol–formaldehyde resins for plywood. In this respect, adhesion of plywood by the first of these with limited formylated lignin addition (up to 20%) has been used extensively in the past, in Canada, for plywood [69,70]. More recently, an LPF resin according to the second approach has been commercialized for plywood containing up to 50% lignin [71], and improvements on this by using ionic liquid-treated lignin to prepare LPF and LP-glyoxal resins have also been reported with encouraging results [72,73,74]. A further new approach for lignin wood adhesives uses pre-methylolated lignin with small amounts of a synthetic PF resin and of isocyanates (PMDIs), the lignin proportions being up to 65% of total solids [75,76]. Some new and rather promising technologies on totally biolignin adhesives have, however, also been developed recently. These are (a) adhesives for particleboard, thin hardboard, and other agglomerate wood panels based on a mix of tannin/hexamine with pre-glyoxalated lignin [46], and (b) similar formulations for high resin content, high-performance agricultural fiber composites [77,78,79,80,81,82,83].

The newest approach was obtained from the research on sodium periodate specific oxidation of carbohydrates pursued in the ambit of the research on soy flour adhesives (see later) [53,54]. In this approach, periodate-specific oxidation of pre-demethylated lignin by cleavage of some bonds of its aliphatic chains at the level of its beta-O-4 bonds and vicinal C–OH groups caused cleavage of the lignin structure, yielding a variety of nontoxic and nonvolatile aldehyde groups in the lignin structure itself. These reacted with the lignin aromatic nuclei to give a lignin adhesive, yielding good bonding results for plywood [84].

**Protein Adhesives**: First of all, it must be noted that excellent wood composite UF resins containing up to 15% of proteins have already been commercially available for quite a long time now, around a decade. These are the AsWood resins for wood panel products. However, this field is dominated by the abundant research work on soy protein and soy flour adhesives. A considerable number of different approaches have been tried with encouraging results. First technologies based on the pre-reaction of soy protein hydrolysate pre-reacted with formaldehyde, and this being mixed with a PF resin and with isocyanate (pMDI) gave encouraging results [85,86]. The same with pre-glyoxalated soy and/or wheat gluten with PF and pMDI [87,88]. An even more interesting, non-traditional system is based on the pre-reaction of the soy protein hydrolysate with maleic anhydride to form an adduct that is then reacted in the panel with polyethylene imine [89,90]. This system also works well, as one company has started using it industrially in the United States, but suffers from the drawback of being expensive. Equally, work on protein–glutaraldehyde adhesives has been published [91].

Three totally new experimental approaches to yield totally biobased soy flour and soy protein wood adhesives have very recently come to the fore. The first is based on the reaction of a hydrolyzable tannin (tannic acid), or a condensed tannin with soy protein isolate and/or soy flour. This gave rather encouraging results for plywood, particleboard, and hardboard [47,92,93,94]. The second unusual approach is by the formation of soy protein isolate-based polyamides formed by the reaction of soy protein isolate with maleic anhydride and hexamethylene diamine [95] as a totally new resin for such an application. The third approach to obtain totally biobased adhesives is even more revolutionary. It is based on the specific oxidation of carbohydrates by sodium periodate. The bonding results using soy flour gave good bonding results for plywood [53]. The periodate treatment was found to cause cleavage of monomeric and polymeric carbohydrates in soy flour, forming a variety of nontoxic and nonvolatile monomeric and oligomeric aldehydes. These reacted with the protein part of the soy flour to give good bonding results [54]. Even more interesting was that periodate oxidation formed aldehyde groups on the soy protein itself, this also contributing to good bonding results [53,54].

**Carbohydrate Adhesives**: Carbohydrates can be used as wood panel adhesives in three main ways: (i) as modifiers of existing PF and UF adhesives, with considerable literature on this traditional approach already existing [96,97,98,99,100,101,102], (ii) by forming degradation compounds which then can be used as adhesives building blocks, and (iii) directly as wood adhesives. The second route leads to furanic resins. Furanic resins, notwithstanding that their basic building blocks, furfuraldehyde and furfuryl alcohol, are derived from acid treatment of the carbohydrates in waste vegetable material, are considered today as purely synthetic resins [103]. This opinion needs to change, as they are real natural-derived bio-resins, and extensively used in applications other than wood composite binders. Appropriate reviews dedicated just to them do exist [103]. However, both compounds are relatively expensive and very dark-colored, and furanic resins have made their industrial mark in fields where their high cost is not a disadvantage. They can be used very successfully for panel adhesives, but the relatively higher toxicity of furfuryl alcohol before it is reacted needs to be addressed if these resins are to be considered for wood composites. In this context, furanic–aldehyde resins for wood composites starting from furfuryl alcohol and nontoxic, nonvolatile aldehydes have also been reported [104].

Several research groups [105,106] have recently described the use of liquefied products from cellulosic materials, literally liquefied wood, which showed good wood adhesive properties. Lignocellulosic and cellulosic materials were liquefied in the presence of sulfuric acid under normal pressure using either phenol or ethylene glycol.

**Figure 2:**
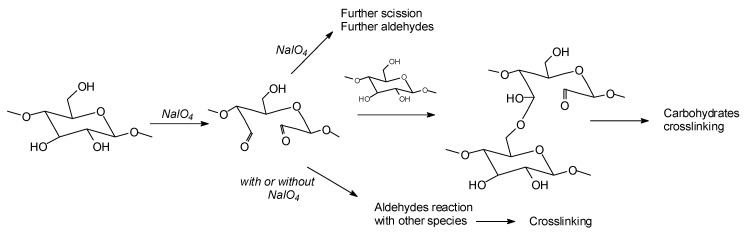
Specific oxidation of carbohydrates by sodium periodate leading to nontoxic and nonvolatile biosourced aldehydes and to different crosslinked products according to the conditions used [54].

The oxidation by periodate ion, resulting in a 1,2-glycol scission, is one of the most widely used reactions in carbohydrate chemistry. The mild reaction and the aqueous solvent conditions for periodate oxidation are particularly apt for use with water-soluble carbohydrates. The development and wide application of the reaction are due to its high degree of selectivity [53,54,107,108,109]. While the periodate specific oxidation reaction and outcome are better known for carbohydrate monomers [54,107] and dimers [54] (Figure 2) such as glucose and sucrose, equivalent reactions are also known for higher carbohydrate oligomers up to cellulose itself [53,54,108,109] (Figure 2). In the case of cellulose and long carbohydrate oligomers alone, the reactions that have been shown to occur are the condensation of the aldehydes formed with other carbohydrate chains to yield crosslinking, leading to solid panels in the case of cellulose (Figure 2) [109].

By increasing the level of oxidation with further periodate in the presence of an aldehyde-reactive species such as a soy protein [53,54], a flavonoid tannin [55], or other reactive species, including lignin, crosslinking can also occur, leading to feasible wood adhesives [53,54,55,84] already described above (Figure 2).

**Figure 3:**
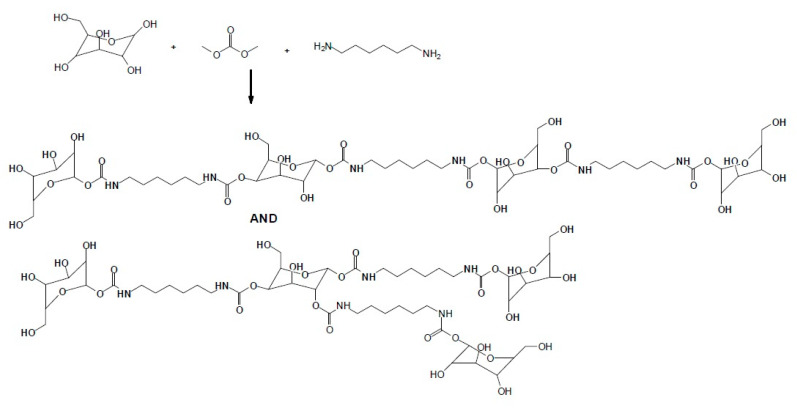
Schematic representation of the formation of non-isocyanate polyurethane (NIPU) oligomers from monosaccharides reacted with dimethyl carbonate and hexamethylene diamine. The two tetramers shown have been identified and are among the multitude of other NIPU oligomers identified [110].

A recent approach to wood composite binders is based on glucose- and sucrose-based non-isocyanate polyurethanes (NIPUs). The finding that glucose and sucrose reacted with dimethyl carbonate (or other cyclic dicarbonates) and a diamine to form NIPU resins [110] (Figure 3) has led to the application of these resins as wood panel composite adhesives [111] with very encouraging results. An equally new approach is the use of the reaction of glucose with maleic anhydride and a diamine to rather form polyamide binders, rather than NIPU, that also yielded wood particleboard binders showing good results [95].

**Figure 4:**
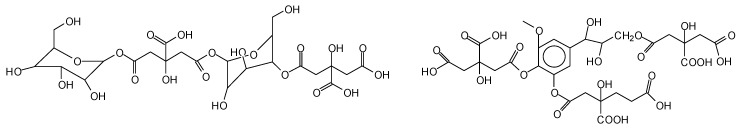
Example of citric acid bridges linking glucoses or holocellulose chains, and actually identified reaction products of citric acid with lignin. The compounds shown have been identified among several others of the same type, showing that both holocellulose and lignin are involved in wood bonding and citric acid bridge-forming by citric acid alone [116].

**Figure 5:**
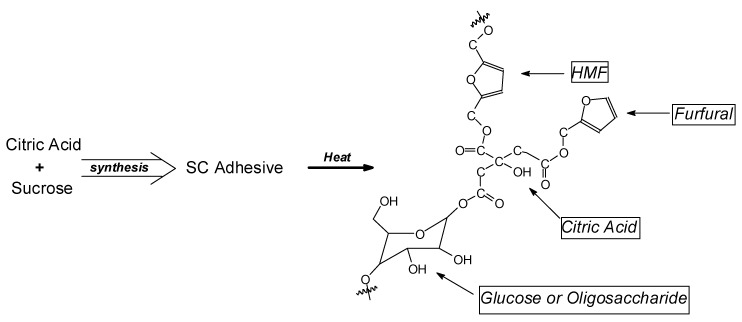
Proposed bridges and possible structures formed by the reaction citric acid with sucrose and with the furfural and hydroxymethyl furfural generated from sucrose by the citric acid treatment in sugar + citric acid adhesives [118].

Equally exciting and revolutionary has been the development of the use of citric acid as a wood composite binder. This is articulated in a number of different approaches, all involving carbohydrates in one manner or another. The concept of citric acid binders for wood composites was first advanced by Japanese researchers yielding very encouraging bonding results [112,113]. The use of citric acid was then extended later to application to wood welding with increased waterproofing of the wood weld line [114] and to bonding of flat veneers such as plywood and LVL [115]. This latter work showed, by chemical analysis, that citric acid was able to function as a binder of the carbohydrates in wood by reacting with them as well as to react with lignin, across two joint veneers [115], with species as in Figure 4 being identified, and was a viable plywood binder. Again, sugar, such as in sucrose + citric acid, was demonstrated as well to be a very viable adhesive for wood composite panels [116,117,118,119] in which not only furfural and hydroxymethyl furfural were formed by the action of the acid on the sugar but also crosslinking of the sugar and the holocellulose carbohydrates by the citric acid was observed (Figure 5), corroborating the same crosslinking with wood carbohydrates, glucose, and with lignin by the action of citric acid directly on wood, as found by Del Menezzi et al. (Figure 4) [115].

### 5.2. Other Bioadhesives for Wood Composites

There are other biomaterials that have been proposed and used for the preparation of bioadhesives. None of the resins presented in this section have reached, at least up to now, industrial trials, but they are interesting and unusual concepts that are worthwhile to pursue and report on.

First, cashew nutshell liquid, mainly composed of cardanol but containing also other compounds. Its dual nature, a resorcinol aromatic ring with an unsaturated fatty acid chain, makes a potential natural raw material for the synthesis of water-resistant resins and polymers. The resorcinol group and/or the double bonds in the chain can be directly used to form hardened networks. Alternatively, more suitable functional groups, such as aldehyde groups and others, can be generated by ozonolysis on the alkenyl chain. The first reaction step yields a cardanol hydroperoxide as major product, that following reduction by glucose or by zinc/acetic acid, yields a high proportion of cardanol-derived aldehyde groups. These crosslink with the aromatic groups of cardanol itself and, thus, a self-condensation of the system, yielding hardened networks [120]. A good review on cardanol thermoset polymers other than for wood composites does exist [121].

Equally, adhesives based on vegetable oils have also been proposed. Encouraging techniques involving unsaturated oils for wood and wood fiber adhesives have been reported [122]. Wheat straw particleboards were made using UF and acrylated epoxidized soy oil (AESO) resins with two resin content levels: 8% and 13%. The physical and mechanical properties of these boards showed that AESO-bonded particleboards have higher physical and mechanical properties than UF-bonded boards, especially regarding internal bonding and thickness swelling [122]. Bioresins based on soybean and other oils have also been mentioned in the literature [123]. These liquid resins were obtained from plant and animal triglycerides by suitably functionalizing the triglyceride with chemical groups (e.g., epoxy, carboxyl, hydroxyl, vinyl, amine, etc.) that render it polymerizable. The reference claims that composites were made using natural fibers such as hemp, straw, flax, and wood in fiber, particle, and flake form, but no results were given. In an older work, an epoxidized oil resin was evaluated as a wood adhesive in composite panels, and it could be tightly controlled through the appropriate selection of triglycerides and polycarboxylic anhydrides [124]. The literature on this resin [123] claims that crosslinking can be varied through the addition of specialized catalysts and several samples were prepared at a range of temperatures (120–180°C) that exhibited high water tolerance, even at elevated temperatures. Their main drawbacks appear to be a slow hot press time and their relatively high cost.

Fungal mycelium bonding is another very new and unusual concept that has been tested and presented for bonding wood panel composites. These new mycelium-based biocomposites (MBBs) were obtained from local agricultural (hemp shives) and forestry (wood chips) byproducts which were bonded together with natural growth of fungal mycelium [125]. As a result, hemp mycocomposites (HMCs) and wood mycocomposites (WMCs) were manufactured and their mechanical, water absorption, and biodegradation properties determined. Compression strength was better for WMCs by about 60% compared to that of HMCs. Water absorption and swelling were relatively high, and these composites were extensively biodegradable, an advantage for certain applications and a disadvantage for others.

Finally, in this category, one can count wood friction welding without adhesives. Rapid friction of two wood surfaces, one against the other, partially melts and mobilizes the intercellular wood material, mainly lignin and xylan hemicelluloses, to form a high-density and high-strength composite interphase formed by intertwined wood fibers and molten material [126,127,128,129]. A basic review on this subject exists [130]. The system works well both as linear welding or rotational dowel welding. Several systems have been recently developed to improve its water resistance [114,131,132]. Linear wood friction welding is an impressive but rather limited technique, as it requires expensive machinery and it can weld only pieces of rather limited length, around one meter. Rotational dowel welding, instead, is very flexible, needing only inexpensive but good quality hand or fixed drills, and presents no limitations of application. It has been and is already being used in small workshops for some biofurniture, assembly of small wood pieces, and room refitting [133,134,135,136,137,138,139,140,141,142]. Some temperature-welded plywood has also been prepared, but its hot press time is far too long to be of industrial significance [143,144].

There are numerous other natural biomaterials, oligomers, and polymers that have been used as binders in fields other than wood composites, but that would be worthwhile to test for wood composites as well. Among these, one can mention humins, isosorbide, itaconic acid, urushiol, and others [145,146,147,148,149].

## 6. New Approaches to Synthetic Adhesives

It must be clearly pointed out that while the environmental concern that predominates today has led to a considerable increase in research and publication on biobased adhesives for wood composites, the research on synthetic adhesives also has made huge progress with a host of new ideas and approaches been thought out and presented. Not all can be presented here, but a few of the more interesting examples are selected. There are two distinct trends in this context: (i) the ongoing improvement, also considerable, of traditional synthetic adhesives, especially in UF resins with, moreover, very valuable new ideas being presented [150,151,152,153,154,155,156] and MUF resins (see later), and (ii) the presentation of partially non-traditional or even totally non-traditional synthetic adhesives, some also with biobased content.

For the first type of approach, the first tendency has been to prepare engineered UF resins of progressively lower molar ratio, at levels much lower than 1:1 [157], which has become rather common in industry today. This was due to the attempt to minimize formaldehyde emissions from wood panels bonded with UF resins. One of the drawbacks of the much lower than 1:1 molar ratio has been identified in the increase in the tendency of the UF resin to form increasingly present crystalline domains upon hardening as a result of hydrogen bonds between linear molecules [153,158,159,160]. At higher molar ratios, the hardened resin is amorphous, affording better adhesion and better bonding performance. The overly high crystallinity drawback was very recently solved, and solved well [153] by blocking the formation of hydrogen bonds using transition metal ion-modified bentonite nanoclay through in situ intercalation and, thus, converting the crystalline domains of the UF resins to amorphous polymers. Addition of 5% nanoclay to the UF yielded in excess of 50% better adhesion, and almost 50% lower formaldehyde emission, thus resulting in a marked improvement in performance with a low level of crystallinity.

In the same trend, the potential introduction of a very acid pH condensation step in the preparation of UF resins, inducing the formation of occasionally considerable amounts of uron (a cyclic intramolecular urea methylene ether) in the UF resins of lower formaldehyde emission has attracted some research interest [149]. This initial work indicated that introduction of such an acid step can lead to UF resins of improved bonding strength, but also of higher post-cure formaldehyde emission. The results also indicated that minimization of the formation of urons yielded better UF resins when the strongly acid condensation step is introduced in the reaction. Subsequent work on the very acid step showed favorite uron formation at pH values higher than 6, and lower than 4 at which the equilibrium urons/dimethylol ureas are shifted in favor of the cyclic uron species [151]. If the pH is slowly shifted from one pH range to the other, the equilibrium shifts in favor of the formation of a majority of methylol urea. A rapid change in pH does not cause this to any great extent. UF resins with high uron proportions showed this structure linked by methylene bridges to urea and other urons, and also as methylol urons, the reactivity of the methylol group of this latter being much lower than that of methylol urea. Thermomechanical analysis (TMA) tests and tests on wood particleboard prepared with uron-rich resins to which urea was added at the end of the reaction yielded bonds of good strength. Equally, mixing a uron-rich resin with a low F/U molar ratio UF resin yielded resins of greater strength than a simple UF of corresponding molar ratio, indicating that UF resins of lower formaldehyde emission with still acceptable strength could be prepared with these resins [151]. Subsequent research on this aspect compared five resins produced in different manners: four via the traditional alkaline–acid process and one using the strongly acid step process [152]. The differences between the syntheses were mainly based on different formaldehyde/urea molar ratios during the synthesis, temperatures, and the number of urea additions. The resins differed in some characteristics, namely percentage of unreacted oligomers, chemical composition, viscosity, and reactivity [152]. The internal bonds of the particleboards bonded with them was similar for all the resins prepared with the alkaline–acid process, but were better than those bonded with the strong acid step; nonetheless, formaldehyde emission appeared to be independent of the type of synthesis used.

Very reactive iminoamino methylene base intermediates (CH_2_=NH–CH_2^+^_) obtained by the decomposition of hexamethylenetetramine (hexamine) [43,44,45] stabilized by the presence of strong anions such as SO_4_^−2^ and HSO_4^−^_, or hexamine sulfate were shown to markedly improve the water and weather resistance of hardened melamine–urea–formaldehyde (MUF) resins used as wood adhesives, and of the wet internal bond strength performance of wood composite boards bonded with them [45,161,162]. The effect was shown to be induced by very small amounts, between 1 and 5 wt % of this material on resin solid content. This strong effect allowed the use of MUF resins of much lower melamine content and also provided good performance of the bonded joints. Because the main effect was also present at the smaller proportion of hexamine as hexamine sulfate, it was not due at all to any increase in the molar ratio of the resin as a consequence of hexamine sulfate addition [45,162]. The effect of hexamine sulfate was closely linked to the strong buffering action it has on MUF resins. Its role is mainly to induce regularity of the reaction and the stability of conditions during resin networking, due to the buffer [161,162]. Shifting of the polycondensation/degradation equilibrium to the left appeared to be the determining factor. This was a consequence of maintaining a higher, constant pH during curing due to the buffer action. The resins are faster-curing than when catalyzed by ammonium sulfate. The effect is valid within the narrow buffering range of pH used for resin hardening. Polycondensation is far too slow to occur at a much higher pH, and degradation is, instead, more predominant at much lower pH. The network formed is then more crosslinked and less tainted by degradation when curing occurs within the correct pH range [45,161,162]. The result is a much better performance of the wood board after water attack. The effects induced by hexamine sulfate are of longer duration than those of other potential buffers. This is due to the hexamine sulfate heat stability under standard hot curing conditions of the resin. Alternate systems were found and shown to have a comparable effect. This approach has already been used to a limited extent in some industrial MUF adhesives.

Of interest is the upgrading of UF, MUF, and PF resins by addition of PMDI (polymeric 4,4′-diphenyl methane diisocyanate) directly in the water-based formaldehyde-based resin glue mix before application to wood [30,31]. This was shown to lead to crosslinking by both formation of the traditional methylene bridges and simultaneously coupled with them by the formation of urethane bridges [30,31]. This is now a currently used industrial adhesive system, especially for UF and MUF resins, and proved to be to be the only system at the time to prepare polyurethane bridges in water. It was based on the stability of PMDI in water for about 5 h before being water-deactivated. Variations of the theme, such as the use of water emulsifiable blocked PMDI [163,164,165] and, more recently, of microencapsulated pMDI [166] have also been tried. They both worked and were attempted to see if an improvement on the original, simpler, system could be achieved. The bonding performance for both is the same as the simpler system, but the stability of the isocyanate in water is much improved affording, perhaps, the preparation of a stable premix of better shelf- and pot-life.

Very recently, the catalytic influence of TiO_2_ in accelerating both synthesis and cure of MF resins has been found [154]. The effect is noticeable and could be due to a variety of causes. There are several possible explanations for this effect. Thus, firstly, Ti is known to form coordination complexes with carbonyl groups (as an aldehyde to pass the C=O to a C–OH, with the intermediate CH_2_–O–TiO_2_, thus an effect of acceleration due to the Ti charge being stronger than that of H^+^. This effect is well known in PF resins, for example. In this case, one should assume that the Ti in TiO_2_ behaves as a divalent ion. With this approach, TiO_2_ might even complex more than one molecule of formaldehyde at the same time, possibly rapidly increasing the number of aldehyde molecules added onto the same or to different NH_2_ groups, also explaining the acceleration of the rate of reaction. The second possibility is the McMurry reaction, where catalysis by Ti joins two carbonyl groups by elimination of the two oxygens, thus from H_2_C=O + O=CH_2_ passing to CH_2_=CH_2_. What would happen, then, is not the formation of just CH_2_=CH_2_ but the reaction of the NH_2_ group of melamine with the C of two formaldehyde molecules and the formation of a –NH–CH_2_=CH_2_–NH– bridge between two molecules of melamine and, thus, two formaldehyde at the time bridging instantly, again explaining the catalysis of the reaction.

The third possibility is that if TiO_2_ forms a stable complex, then the initial attack of the aldehyde on the melamine will be fast, but after that, all will remain blocked [26,27,28]. It has been proven that this is not the case as the reaction accelerates. It means that if complexes are formed, the rate of exchange in solution is very rapid [167] as the complex is not stable (and the TiO_2_ complexes cannot be very stable, otherwise they could not function as catalysts in a number of organic chemistry reactions) and, thus, only the effect of the initial fast attack remains, hence, the acceleration in rate [26,27,28].

Lastly a further explanation might well be valid, thus that once blocked the reaction of the aldehyde with the melamine the bridges forming in the resin are instead through the Ti itself if the complexes formed are stable. First, as –NH–CH_2_O–Ti–OCH_2_–NH– with the other two valences of the Ti have been linked in the same way to other NH–CH_2_–O groups, thus forming a tridimensional knot, this would easily explain the strong acceleration. It is very possible that several, if not all, of these mechanisms are at work, although to a different extent.

Having discussed interesting advances in wood composite aminoplastic adhesives, one has to say that progress has been equally impressive in PF adhesives in the last two decades. Just two cases are reported here, although there are others that are also of interest.

The first regards the curing and reaction rate acceleration mechanism of the resins for wood composites induced by certain esters (also called the “alpha-set” in jargon). The concept of ester acceleration was initially promoted by the Borden company in the early 1950s, but applied in a different manner to foundry core binders, not to wood composites [168]. The approach was rather modified to be adapted to wood composite binders with propylene carbonate and triacetin (glycerol triacetate) being, finally, the two esters retained for wood composite binders [169,170,171,172,173]. Controversy followed, with some research groups claiming that nothing was occurring [174,175], and others claiming that it was the same type of acceleration being caused by inorganic carbonates, such as sodium and potassium carbonates [176], which was disproved [172], while other groups instead also observed some structural resin modifications [173,177,178,179], with the controversy finally being resolved by isolating and identifying the structural modifications caused to the resin by the esters [180].

The mechanism was found to involve the phenate ion of the resin to apparently yield a carbonyl or carboxyl group attached to the aromatic ring. Either directly or by subsequent rapid rearrangement after the initial attack on the ortho site, these C=O groups were found on sites different from the ortho site. The appearance determined from NMR shift calculation indicated preferential positioning or repositioning to the para site and, surprisingly, to the meta sites of the phenolic ring. The shifts of these C=O groups correspond to those of an anhydride and to no other intermediate structures previously thought of. Anhydride-like bridges were clearly shown by MALDI-TOF mass spectrometry to contribute to oligomer structures in which linkages between phenol rings were mixed methylene bridges and anhydride bridges. These structures appeared to be temporary, possibly due to the instability of the anhydride bridges; hence, they were in small proportions at any given moment of the reaction. ^13^C NMR and MALDI-TOF analysis clearly indicated that these structures were, at some moment, an integral part of the structure of the liquid resin and that they existed parallel to the methylene bridges pertaining to a normal PF resin structure [180]. While the complexity and unusualness of the mechanism is interesting, the main importance of ester acceleration is to be able to use PF resins with simpler and shorter manufacture time and of much faster hot press time of the wood composite, a real industrial advantage.

Low-condensation phenol–formaldehyde (PF) resins co-reacted under alkaline conditions with up to 42% molar urea on phenol during resin preparation yielded PUF resins capable of faster hardening times than equivalent pure PF resins prepared under identical conditions and presented better performance than the latter [181,182]. The water resistance of the prepared PUF resins seemed comparable to pure PF resins when used as adhesives for wood composite panels. Part of the urea is copolymerized to yield the alkaline PUF resin, but unreacted urea was still present in the resin, especially at the higher levels of urea addition. Increase of the initial formaldehyde to phenol molar ratio considerably decreased the proportion of unreacted urea and increased the proportion of PUF resin. The copolymerized urea functions as a prebranching molecule in the forming, hardening resin network. PUF resins are capable of further noticeable curing acceleration by addition of ester accelerators; namely, glycerol triacetate (triacetin), to reach gel times as fast as those characteristic of catalyzed aminoplastic resins, but with wet strength values of the wood composites bonded with them, characteristic of exterior PF resins. Synergy between the relative amounts of copolymerized urea and ester accelerator occurs at the lower levels of the two additives. However, this synergy decreases at the higher percentages of urea and triacetin. The relative performance of PUF adhesives was checked by preparation of wood particleboard, and the capability of the accelerated PUF resins to achieve press times as fast as those of aminoplastic (UF and others) resins has been confirmed [181,182]. This system has been in industrial utilization, to a moderate extent, for more than a decade.

For the non-traditional synthetic adhesives, the (at first shocking) concept is that urea–formaldehyde can also be classed as a bioadhesive. Urea is bioderived from nitrogen in the air, but while formaldehyde exists in nature, its industrial production is not “bio” at all. Thus, there is interest in eliminating formaldehyde through replacement with something less or nontoxic and, especially, nonvolatile (to eliminate formaldehyde emission). The first attempts to solve this problem led to the preparation of urea–glyoxal (UG) resins for wood composite adhesives [183,184]. Urea–glyoxal resins are already known and used in the textile industry, but the formulations needed to be extensively changed for wood composites. The problem encountered was that UG resins are much slower curing in hot-pressed than UF resins, their energy of activation for curing being markedly higher. The first approach to solve this drawback was to prepare and use melamine–glyoxal (MG) resins, melamine being much more reactive with aldehydes than urea. This yielded an improvement to the point that these MG resins could at least be used for paper impregnation, the resin being in direct contact with the hot platen of the press and, thus, at higher temperature [185]. However, while faster, they were still too slow as adhesives for bonding wood panel composites. The breakthrough to solve these very limiting drawbacks came with the introduction of ionic liquids (ILs) as hardeners of UG resins. These markedly decreased the energy of activation of curing of UG adhesives and the resultant IL UG adhesives were used to prepare wood particleboards at pressing times and with results comparable with UF resins [186]. The approach then progressed to IL MG and, finally, to IL melamine–glyoxal–glutaraldehyde (IL MGG’) adhesives that gave a very acceptable performance as adhesives for wood composites [187].

Equally noticeable in this context is the renewed trend and renewed interest in resins based on urea furfural and urea–furfuryl alcohol having become, again, a basis of study, notwithstanding that these are old technologies used for other applications [188,189]. New, however, is the interest in hydroxymethyl furfural, with urea–hydroxymethyl furfural [190] and phenol–hydroxymethyl furfural [191] as well as tannin–hydroxymethyl furfural [192]. While these are acceptable for phenol and natural polyphenols such as tannins, that are anyhow dark adhesives, the natural developing dark colors of the furanic materials constitute a serious commercial drawback for urea resins that are traditionally transparent or white. Resins based on furfuryl alcohol + an aldehyde have also been tested with encouraging results for plywood bonding, these being furfuryl–alcohol formaldehyde, furfuryl alcohol–glyoxal, and furfuryl alcohol–glutaraldehyde [104].

**Figure 6:**
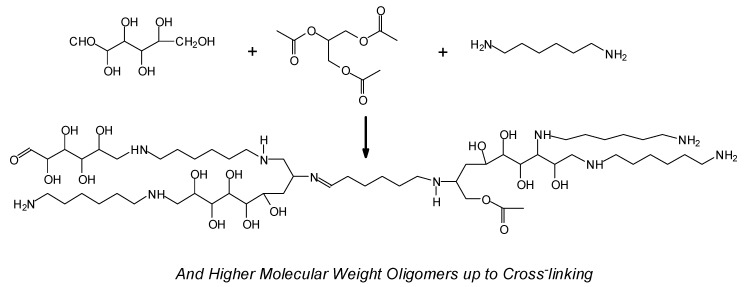
Example of a mixed higher molecular weight oligomer leading to adhesive crosslinking formed, among others, in the new adhesive system derived by the reaction of glucose or sucrose with triacetin and hexamethylene diamine [194].

Even more different approaches to mainly synthetic thermoset adhesives have been tried and presented. For example, glucose or sucrose reacted with glycerol triacetate and a diamine yielded a variety of mixed oligomers (Figure 6) and a very acceptable wood adhesive for plywood. The system was found by pure chance, but it worked, and the oligomers and polymers formed were identified and characterized [193].

## 7. Thermoplastics as Binders for Wood Composites

Very promising directions for the bonding of plywood and LVL based on environmentally friendly or even recycled products, is the field where thermoplastics (polyethylene, polypropylene, poly(vinyl chloride), and their copolymers) are used as the wood composite binders. Such thermoplastic polymers, as a substitute for conventional UF and PF thermosetting adhesives, can be used for veneer bonding in various forms, such as textile fiber waste (i.e., polyurethane, polyamide-6), recycled plastic shopping bags, or film. This area constitutes an area of major research effort especially in recovering and reusing waste polymeric materials. One example of this is the use of waste polyurethanes for the binding of particleboard and plywood [195,196]. There are excellent reviews on this wide field and the reader is referred to these for a more in-depth look of this area [197,198,199,200].

Furthermore, dry adhesive film, in particular, is now used for plywood as it is simpler to apply than wet adhesives; all of the untidy and unpleasant mixing and spreading operations in wet gluing are thus wholly removed from the factory floor by the use of such dry adhesive films. The dry adhesive film contains, in each square meter of surface, precisely the same quantity of adhesive, equal quality, uniform composition, yielding exactly the same bond strength and the same standard thickness [194].

## 8. Conclusions

The field of wood composites is a huge field of research of vast economic importance, fast moving both in the conception of newer composites but even more in the conception and development of newer and sometimes even revolutionary types of binders for them. While the wood composite industry, mainly for reason of supply, is still dominated by traditional oil-derived adhesives, both in these fields as well as in the strongly upcoming field of biobased adhesives, there has been almost incredible progress as well as developments dictated by the intellectual ferment induced by a number of outside constraints. These are the stricter government regulations to reduce and even eliminate formaldehyde and other materials that are to some extent toxic, consumer awareness and the consequent drive of industry to favor more environment-friendly materials and, finally, the drive of industry to decrease or even eliminate their dependence on petrochemicals, due to the real or imagined future decrease of oil reserves with its consequent increase in the price of raw materials for purely traditionally manufactured wood binders. As it stands, what presented in this review is only a brief overview of what has happened and is happening in this field, with possibly lesser or more difficult to implement approaches not being mentioned or not mentioned enough. Progress in this fascinating field of primary economic importance has been accelerating, and the number of new ideas, approaches, and new proposed binder systems is continuously increasing, providing a glimpse of an exciting and interesting research future.

## References

[1] Pizzi A., Belgacem M.N., A.Pizzi A. (2017). Wood and Fiber Panels Technology. Lignocellulosic Fibers and Wood Handbook: Renewable Materials for to-Day’s Environment.

[2] Geimer R.L., Mahoney R.J., Loehnertz S.P., Meyer R.W. (1985). Influence of Processing Induced Damage on the Strength of Flakes and Flakeboards.

[3] Maloney T.M. (1993). Modern Particleboard & Dry-Process Fiberboard Manufacturing.

[4] Mantanis G., Athanassiadou E., Barbu M., Wijnendaele K. (2018). Adhesive systems used in the European particleboard, MDF and OSB industries. Wood Mater. Sci. Eng..

[5] Pizzi A. (1983). Wood Adhesives Chemistry and Technology.

[6] Pizzi A., Pizzi A., Mittal K.L. (2003). Urea-formaldehyde adhesives. Handbook of Adhesive Technology.

[7] Pizzi A., Pizzi A., Mittal K.L. (2017). Urea and melamine aminoresin adhesives. Handbook of Adhesive Technology.

[8] Pizzi A. (1994). Advanced Wood Adhesives Technology.

[9] Pizzi A., Pizzi A., Mittal K.L. (2003). Melamine-formaldehyde adhesives. Handbook of Adhesive Technology.

[10] Pizzi A., Pizzi A., Mittal K.L. (2017). Phenolic resin adhesives. Handbook of Adhesive Technology.

[11] Pizzi A., Pizzi A., Mittal K.L. (2003). Phenolic resin adhesives. Handbook of Adhesive Technology.

[12] Pizzi A., Pizzi A., Mittal K.L. (2003). Resorcinol adhesives. Handbook of Adhesive Technology.

[13] Frazier C.E., Pizzi A., Mittal K.L. (2003). Isocyanate wood binders. Handbook of Adhesive Technology.

[14] Lay D.G., Cranley P., Pizzi A., Pizzi A., Mittal K.L. (2017). Polyurethane adhesives. Handbook of Adhesive Technology.

[15] Pizzi A., Pizzi A. (1983). Tannin-based wood adhesives. Wood Adhesives Chemistry and Technology.

[16] Pizzi A. (1978). Wattle-based adhesives for exterior grade particleboard. For. Prod. J..

[17] Pizzi A., Scharfetter H. (1978). The chemistry and development of tannin-based wood adhesives for exterior plywood. J. Appl. Polym. Sci..

[18] Valenzuela J., von Leyser E., Pizzi A., Westermeyer C., Gorrini B. (2012). Industrial production of pine tannin-bonded particleboard and MDF. Eur. J. Wood Prod..

[19] Pizzi A. (1979). Glue blenders effect on particleboard using wattle tannin adhesives. Holzforsch. Holzverwert..

[20] Pizzi A. (1977). Hot-setting tannin-urea-formaldehyde exterior wood adhesives. Adhes. Age.

[21] Custers P.A.J.L., Rushbrook R., Pizzi A., Knauff C.J. (1979). Industrial applications of wattle-tannin/urea-formaldehyde fortified starch adhesives for damp-proof corrugated cardboard. Holzforsch. Holzverwert..

[22] Pizzi A., Roux D.G. (1978). The chemistry and development of tannin-based weather- and boil-proof cold-setting and fast-setting adhesives for wood. J. Appl. Polym. Sci..

[23] Pizzi A., Rossouw D.D.T., Knuffel W., Singmin M. (1980). “Honeymoon” phenolic and tannin-based fast setting adhesive systems for exterior grade fingerjoints. Holzforsch. Holzverwert..

[24] Pizzi A., Cameron F.A. (1984). Fast-set adhesives for glulam. For. Prod. J..

[25] Mansouri H.R., Pizzi A., Fredon E. (2009). Honeymoon fast-set adhesives for glulam/fingerjoints of higher natural materials content. Eur. J. Wood. Prod..

[26] Pizzi A. (1979). Phenolic resins by reactions of coordinated metal ligands. J. Polym. Sci. Polym. Lett..

[27] Pizzi A. (1979). Phenol and tannin-based adhesive resins by reactions of coordinated metal ligands, Part 1: Phenolic chelates. J. Appl. Polym. Sci..

[28] Pizzi A. (1979). Phenol and tannin-based adhesive resins by reactions of coordinated metal ligands, Part II: Tannin adhesives preparation, characteristics and application. J. Appl. Polym. Sci..

[29] von Leyser E., Pizzi A. (1990). The formulation and commercialization of glulam pine tannin adhesives in Chile. Holz Roh-und Werkst..

[30] Pizzi A., Walton T. (1992). Non-emulsifiable, water-based diisocyanate adhesives for exterior plywood, Part 1: Novel reaction mechanisms and their chemical evidence. Holzforschung.

[31] Pizzi A., Valenzuela J., Westermeyer C. (1993). Non-emulsifiables, water-based, diisocyanate adhesives for exterior plywood, Part 2: Industrial application. Holzforschung.

[32] Böhm R., Hauptmann M., Pizzi A., Friederich C., Laborie M.-P. (2016). The chemical, kinetic and mechanical characterization of Tannin-based adhesives with different crosslinking systems. Int. J. Adhes. Adhes..

[33] Santiago-Medina F.J., Foyer G., Pizzi A., Calliol S., Delmotte L. (2016). lignin-derived non-toxic aldehydes for ecofriendly tannin adhesives for wood panels. Int. J. Adhes. Adhes..

[34] Ndiwe B., Pizzi A., Tibi B., Danwe R., Konai N., Amirou S. (2019). African tree bark exudate extracts as biohardeners of fully biosourced thermoset tannin adhesives for wood panels. Ind. Crops Prod..

[35] Pizzi A., Meikleham N., Dombo B., Roll W. (1995). Autocondensation-based, zero-emission, tannin adhesives for particleboard. Holz Roh-und Werkst..

[36] Meikleham N., Pizzi A., Stephanou A. (1994). Induced accelerated autocondensation of polyflavonoid tannins for phenolic polycondensates, Part 1: ^13^C NMR, ^29^Si NMR, X-ray and polarimetry studies and mechanism. J. Appl. Polym. Sci..

[37] Pizzi A., Meikleham N., Stephanou N. (1995). Induced accelerated autocondensation of polyflavonoid tannins for phenolic polycondensates—Part II: Cellulose effect and application. J. Appl. Polym. Sci..

[38] Garcia R., Pizzi A., Merlin A. (1997). Ionic polycondensation effects on the radical autocondensation of polyflavonoid tannins-An ESR study. J. Appl. Polym. Sci..

[39] Garcia R., Pizzi A. (1998). Polycondensation and autocondensation networks in polyflavonoid tannins, Part 1: Final networks. J. Appl. Polym. Sci..

[40] Garcia R., Pizzi A. (1998). Polycondensation and autocondensation networks in polyflavonoid tannins, Part 2: Polycondensation vs. autocondensation. J. Appl. Polym. Sci..

[41] Garcia R., Pizzi A. (1998). Cross-linked and entanglement networks in thermomechanical analysis of polycondensation resins. J. Appl. Polym. Sci..

[42] Pichelin F., Nakatani M., Pizzi A., Wieland S., Despres A., Rigolet S. (2006). Structural beams from thick wood panels bonded industrially with formaldehyde free tannin adhesives. For. Prod. J..

[43] Kamoun C., Pizzi A. (2000). Mechanism of hexamine as a non-aldehyde polycondensation hardener, Part 1: Hexamine decomposition and reactive intermediates. Holzforsch. Holzverwert..

[44] Kamoun C., Pizzi A. (2000). Mechanism of hexamine as a non-aldehyde polycondensation hardener, Part 2: Recomposition of intermediate reactive compound. Holzforsch. Holzverwert..

[45] Kamoun C., Pizzi A., Zanetti M. (2003). Upgrading of MUF resins by buffering additives—Part 1: Hexamine sulphate effect and its limits. J. Appl. Polym. Sci..

[46] Navarrete P., Mansouri H.R., Pizzi A., Tapin-Lingua S., Benjelloun-Mlayah B., Rigolet S. (2010). Synthetic-resin-free wood panel adhesives from low molecular mass lignin and tannin. J. Adhes. Sci. Technol..

[47] Ghahri S., Pizzi A., Mohebby B., Mirshoktaie A., Mansouri H.R. (2018). Soy-based, tannin-modified plywood adhesives. J. Adhes..

[48] Abdullah U.H.B., Pizzi A. (2013). Tannin-Furfuryl alcohol wood panel adhesives without formaldehyde. Eur. J. Wood Prod..

[49] Ballerini A., Despres A., Pizzi A. (2005). Non-toxic, zero-emission tannin-glyoxal adhesives for wood panel. Holz Roh-und Werkst..

[50] Trosa A., Pizzi A. (2001). A no-aldehyde emission hardener for tannin-based wood adhesives. Holz Roh-und Werkst..

[51] Grigsby W.J., McIntosh C.D., Warnes J.M., Suckling I.D., Anderson C.R. (2008). Adhesives. U.S. Patent.

[52] Trosa A., Pizzi A. (1998). Industrial hardboard and other panels binder from tannin/furfuryl alcohol in absence of formaldehyde. Holz Roh-und Werkst..

[53] Frihart C.R., Lorenz L. (2019). Specific oxidants improve the wood bonding strength of soy and other plant flours. J. Polym. Sci. A Polym. Chem..

[54] Frihart C.R., Pizzi A., Xi X., Lorenz L. (2019). Reactions of Soy flour and Soy protein by non-volatile aldehydes generation by specific oxidation. Polymers.

[55] Xi X., Pizzi A., Frihart C.R., Lorenz L., Gerardin C. (2020). Tannin plywood adhesives by non-volatile aldehydes generation from specific oxidation of mono- and disaccharides. Int. J. Adhes. Adhes..

[56] Nimz H.H., Pizzi A. (1983). Lignin-based adhesives. Wood Adhesives Chemistry and Technology.

[57] Blanchet P., Cloutier A., Riedl B. (2000). Particleboard made from hammer milled black spruce bark residues. Wood Sci. Technol..

[58] Lopez-Suevos F., Riedl B. (2003). Effects of Pinus pinaster bark extracts content on the cure properties of tannin-modified adhesives and on bonding of exterior grade MDF. J. Adhes. Sci. Technol..

[59] Kim S., Kim H.-J. (2003). Curing behaviour and viscoelastic properties of pine and wattle tannin-based adhesives studied by dynamic mechanical thermal analysis and FT-IR-ATR spectroscopy. J. Adhes. Sci. Technol..

[60] Calvé L.R. (1999). Fast cure and pre-cure resistant cross-linked phenol-formaldehyde adhesives and methods of making same. Canada Patent.

[61] Shimatani K., Sono Y., Sasaya T. (1994). Preparation of moderate-temperature setting adhesives from softwood kraft lignin. Part 2. Effect of some factors on strength properties and characteristics of lignin-based adhesives. Holzforschung.

[62] Gardner D., Sellers T. (1986). Formulation of a lignin-based plywood adhesive from steam-exploded mixed hardwood lignin. For. Prod. J..

[63] Newman W.H., Glasser W.G. (1985). Engineering plastics from lignin-XII. Synthesis and performance of lignin adhesives with isocyanate and melamine. Holzforschung.

[64] Azarov V.I., Koverniskii N.N., Zaitseva G.V. (1985). Izvestjia Vysshikh Uchnykh Zavedenii. Lesnai Zhurna.

[65] Viikari L., Hase A., Quintus-Leina P., Kirsi K., Tuominen S., Gaedda L. (1999). Lignin Based Adhesives and a Process for the Preparation Thereof. European Patent.

[66] Kharazipour A., Haars A., Shekholeslami M., Hüttermann A. (1991). Enzymgebundene holzwerkstoffe auf der basis von lignin und phenoloxidasen. Adhäsion.

[67] Kharazipour A., Mai C., Hüttermann A. (1998). Polyphenols for compounded materials. Polym. Degrad. Stabil..

[68] Antov P., Savov V., Mantanis G.I., Neykov N. (2020). Medium-density fibreboards bonded with phenol-formaldehyde resin and calcium lignosulfonate as an eco-friendly additive. Wood Mat. Sci. Eng..

[69] Calvé L.R. Phenolic-lignin wood adhesives for plywood. Proceedings of the 19th IUFRO World Congress.

[70] Calvé L.R. (1992). Fast Cure and Pre-Cure Resistant Cross-Linked Phenol-Formaldehyde Adhesives and Methods of Making Same. US Patent.

[71] Valkonen S. Lignin-based binders: An industrial reality, latest developments. Proceedings of the International Conference on Wood Adhesives.

[72] Younesi-Kordkheili H., Pizzi A. (2019). Some Properties of Particleboard Panels Bonded with Phenol- Lignin- Glyoxal Resin. J. Adhes..

[73] Younesi-Kordkheili H., Pizzi A. (2018). Properties of plywood panels bonded with ionic liquid-modified lignin–phenol–formaldehyde resin. J. Adhes..

[74] Younesi-Kordkheili H., Pizzi A., Hornabakhsh-Raouf A., Nemati F. (2017). The Effect of Modified Soda Bagasse Lignin by Ionic Liquid on Properties of Urea-Formaldehyde Resin as Wood Adhesive. J. Adhes..

[75] El Mansouri N.E., Pizzi A., Salvado J. (2007). Lignin-based polycondensation resins for wood adhesives. J. Appl. Polym. Sci..

[76] El Mansouri N.E., Pizzi A., Salvado J. (2007). Lignin-based wood panel adhesives without formaldehyde. Holz Roh-und Werkst..

[77] Pizzi A., Kueny R., Lecoanet F., Massetau B., Carpentier D., Krebs A., Loiseau F., Molina S., Ragoubi M. (2009). High resin content natural matrix-natural fibre biocomposites. Ind. Crops Prod..

[78] Mansouri H.R., Navarrete P., Pizzi A., Tapin-Lingua S., Benjelloun-Mlayah B., Rigolet S. (2011). Synthetic-resin-free wood panel adhesives from low molecular mass lignin and tannin. Eur. J. Wood Prod..

[79] Navarrete P., Mansouri H.R., Pizzi A., Tapin-Lingua S., Benjelloun-Mlayah B., Pasch H., Rode K., Delmotte L., Rigolet S. (2012). Low formaldehyde emitting biobased wood adhesives manufactured from mixtures of tannin and glyoxalated lignin. J. Adh. Sci. Technol..

[80] Bertaud F., Tapin-Lingua S., Pizzi A., Navarrete P., Petit-Conil M. (2012). Development of green adhesives for fibreboards manufacturing, using tannins and lignin from pulp mill residues. Cellul. Chem. Technol..

[81] Sauget A., Nicollin A., Pizzi A. (2013). Fabrication and mechanical analysis of mimosa tannin and commercial flax fibers biocomposites. J. Adhes. Sci. Technol..

[82] Zhu J., Abhyanker H., Njuguna J., Perreux D., Thiebaud F., Chapelle D., Pizzi A., Sauget A., Nicollin A. (2013). Tannin-based flax fibre-reinforced bio-composites for structural application in Superlight Electric Vehicles. Ind. Crops Prod..

[83] Nicollin A., Li X., Girods P., Pizzi A., Rogaume Y. (2013). Fast pressing composite using tannin-furfuryl alcohol resin and vegetal fibers reinforcement. J. Renew. Mater..

[84] Chen X., Xi X., Pizzi A., Fredon E., Du G., Gerardin C. (2020). Oxidized Demethylated Lignin as a Bio-Based Adhesive for Wood Bonding. J. Adhes..

[85] Wescott J.M., Frihart C.R., Lorenz L. (2006). Durable soy-based adhesives. Proceedings Wood Adhesives 2005.

[86] Lorenz L., Frihart C.R., Wescott J.M. (2006). Analysis of soy flour/phenol-formaldehyde adhesives for bonding wood. Wood Adhesives 2005: November 2–4, 2005... San Diego, California.

[87] Amaral-Labat G.A., Pizzi A., Goncalves A.R., Celzard A., Rigolet S. (2008). Environment-friendly soy flour-based resins without formaldehyde. J. Appl. Polym. Sci..

[88] Lei H., Pizzi A., Navarrete P., Rigolet S., Redl A., Wagner A. (2010). Gluten protein adhesives for wood panels. J. Adhesion Sci. Technol..

[89] Liu Y., Li K. (2002). Chemical modification of soy protein for wood adhesives. Macromol. Rapid Comm..

[90] Liu K., Li K. (2007). Development and characterization of adhesives from soy protein for bonding wood. Int. J. Adhes. Adhes..

[91] Xi X., Pizzi A., Gerardin C., Liao J., Amirou S., Abdalla S. (2019). Glutaradehyde-wheat protein-based adhesives for plywood. J. Adhes..

[92] Lagel M.C., Pizzi A., Redl A. (2014). Phenol-wheat protein-formaldehyde adhesives for wood-based panels. ProLigno.

[93] Ghahri S., Pizzi A., Mohebby B., Mirshoktaie A., Mansouri H.R. (2018). Improving Water Resistance of Soy-Based Adhesive by Vegetable Tannin. J. Polym. Environ..

[94] Ghahri S., Pizzi A. (2018). Improving Soy-Based Adhesives for Wood Particleboard by Tannins Addition. Wood Sci. Technol..

[95] Xi X., Pizzi A., Gerardin C., Chen X., Amirou S. (2020). Soy Protein Isolate based Polyamides as Wood Adhesives. Wood Sci. Technol..

[96] Conner A.H., River B.H., Lorenz L.F. (1986). Carbohydrates-modified PF resins for wood panels. J. Wood Chem. Technol..

[97] Conner A.H., Lorenz L.F., River B.H. (1989). Carbohydrate-modified PF resins formulated at neutral conditions. ACS Symp. Ser..

[98] Moubarik A., Pizzi A., Charrier F., Allal A., Badia M.-A., Mansouri H.R., Charrier B. (2013). Mechanical characterization of industrial particleboard panels glued with cornstarch- mimosa tannin- urea formaldehyde resins. J. Adhes. Sci. Technol..

[99] Moubarik A., Mansouri H.R., Pizzi A., Charrier F., Allal A., Charrier B. (2013). Corn flour-mimosa tannin-based adhesives without formaldehyde for interior particleboard production. Wood Sci. Technol..

[100] Moubarik A., Allal A., Pizzi A., Charrier F., Charrier B. (2010). Characterization of a formaldehyde-free cornstarch-tannin wood adhesives for interior plywood. Eur. J. Wood Prod..

[101] Moubarik A., Charrier B., Allal A., Charrier F., Pizzi A. (2010). Development and optimisation of a new formaldehyde-free cornstarch and tannin adhesive. Eur. J. Wood Prod..

[102] Moubarik A., Pizzi A., Allal A., Charrier F., Charrier B. (2009). Cornstarch and tannin in phenol-formaldehyde resins for plywood production. Ind. Crops Prod..

[103] Belgacem M.N., Gandini A., Pizzi A., Mittal K.L. (2003). Furan-based adhesives. Handbook of Adhesive Technology.

[104] Xi X., Wu Z., Pizzi A., Gerardin C., Lei H., Du G. (2018). Furfuryl alcohol-aldehyde plywood adhesive resins. J. Adhes..

[105] Alma M.H., Yoshioka M., Yao Y., Shiraishi N. (1998). Preparation of sulfuric acid-catalyzed phenolated wood resin. Wood Sci. Technol..

[106] Alma M.H., Yoshioka M., Yao Y., Shiraishi N. (1996). The preparation and flow properties of HC1 catalyzed phenolated wood and its blends with commercial novolak resin. Holzforschung.

[107] Bobbitt J.M. (1956). Periodate oxidation of carbohydrates. Adv. Carbohydr. Chem..

[108] Guigo N., Mazeau K., Putaux J.L., Heux L. (2014). Surface modification of cellulose microfibrils by periodate oxidation and subsequent reductive amination with benzylamine: A topochemical study. Cellulose.

[109] Codou A., Guigo N., Heux L., Sbirrazzuoli N. (2015). Partial periodate oxidation and thermal cross-linking for the processing of thermoset all-cellulose composites. Compos. Sci. Technol..

[110] Xi X., Pizzi A., Delmotte L. (2018). Isocyanate-free Polyurethane Coatings and Adhesives from Mono- and Di-Saccharides. Polymers.

[111] Xi X., Wu Z., Pizzi A., Gerardin C., Lei H., Zhang B., Du G. (2019). Non-Isocyanate Polyurethane Adhesive from sucrose used for particleboard. Wood Sci. Technol..

[112] Umemura K., Ueda T., Munawar S., Kawai S. (2012). Application of citric acid as natural adhesive for wood. J. Appl. Polym. Sci..

[113] Umemura K., Kawai S. (2015). Development of Wood-Based Materials Bonded with Citric Acid. For. Prod. J..

[114] Amirou S., Pizzi A., Delmotte L. (2017). Citric acid as waterproofing additive in butt joints linear wood welding. Eur. J. Wood Prod..

[115] Del Menezzi C., Amirou S., Pizzi A., Xi X., Delmotte L. (2018). Reactions with Wood Carbohydrates and Lignin of Citric Acid as a Bond Promoter of Wood Veneer Panels. Polymers.

[116] Umemura K., Sugihara O., Kawai S. (2013). Investigation of a new natural adhesive composed of citric acid and sucrose for particleboard. J. Wood Sci..

[117] Widyorini R., Nugraha P., Rahman M., Prayitno T. (2016). Bonding ability of a new adhesive composed of citric acid-sucrose for particleboard. BioResources.

[118] Zhao Z., Sakai S., Chen Z., Zhu N., Huang C., Sun S., Zhang M., Umemura K., Yong X. (2019). Further Exploration of Sucrose–Citric Acid Adhesive: Investigation of Optimal Hot-Pressing Conditions for Plywood and Curing Behavior. Polymers.

[119] Sun S., Zhao Z., Umemura K. (2019). Further Exploration of Sucrose-Citric Acid Adhesive: Synthesis and Application on Plywood. Polymers.

[120] Tomkinson J., Dunky M., Pizzi A., Van Leemput M. (2002). Adhesives based on natural resources. Wood Adhesion and Glued Products: Wood Adhesives.

[121] Jial P., Song F., Li Q., Xia H., Shu X., Zhou Y. (2019). Recent Development of Cardanol Based Polymer Materials: A Review. J. Renew. Mat..

[122] Tasooji M., Tabarsa T., Khazaeian A., Wool R.P. (2010). Acrylated epoxidised soy oil as an alternative to urea-formaldehyde in making wheat sraw particleboard. J. Adhes. Sci. Technol..

[123] Wool R.P. (1998). Proceedings of the second European Panel Products Symposium.

[124] Miller R., Shonfeld U. (2002). Company Literature.

[125] Zimele Z., Irbe I., Grinins J., Bikovens O., Verovkins A., Bajare D. (2020). Novel Mycelium-based Biocomposites (MBB) as Building Materials. J. Renew. Mat..

[126] Gfeller B., Zanetti M., Properzi M., Pizzi A., Pichelin F., Lehmann M., Delmotte L. (2003). Wood bonding by vibrational welding. J. Adhes. Sci. Technol..

[127] Pizzi A., Leban J.-M., Kanazawa F., Properzi M., Pichelin F. (2004). Wood dowels bonding by high speed rotation welding. J. Adhes. Sci. Technol..

[128] Kanazawa F., Pizzi A., Properzi M., Delmotte L., Pichelin F. (2005). Influence parameters in wood dowels welding by high speed rotation. J. Adhes. Sci. Technol..

[129] Mansouri M., Leban J.-M., Pizzi A. (2010). End-grain butt joints obtained by friction welding of high density eucalyptus wood. Wood Sci. Technol..

[130] Leban J.M., Pizzi A., Properzi M., Pichelin F., Gelhaye P., Rose C. (2005). Wood Welding. A challenging alternative to conventional wood gluing. Scand. J. For. Res..

[131] Pizzi A., Mansouri H.R., Leban J.M., Delmotte L., Omrani P., Pichelin F. (2011). Enhancing the exterior performance of wood linear and rotational welding. J. Adhes. Sci. Technol..

[132] Pizzi A., Zhou X., Navarrete P., Segovia C., Mansouri H.R., Placentia Pena M.I., Pichelin F. (2013). Enhancing water resistance of welded dowel wood joints by acetylated lignin. J. Adhes. Sci. Technol..

[133] Bocquet J.-F., Pizzi A., Resch L. (2007). Full-scale (Industrial) wood floor assembly and structures by welded through dowels. Holz Roh-und Werkst..

[134] Bocquet J.F., Pizzi A., Despres A., Mansouri H.R., Resch L., Michel D., Letort F. (2007). Wood joints and laminated wood beams assembled by mechanically welded wood dowels. J. Adhes. Sci. Technol..

[135] Segovia C., Pizzi A. (2009). Performance of dowel-welded wood furniture linear joints. J. Adhes. Sci. Technol..

[136] Segovia C., Pizzi A. (2009). Performance of dowel-welded T-joints for wood furniture. J. Adhes. Sci. Technol..

[137] Oudjene M., Khalifa M., Segovia C., Pizzi A. (2010). Application of numerical modelling to dowel-welded wood joints. J. Adhes. Sci. Technol..

[138] Segovia C., Renaud A., Pizzi A. (2010). Performance of dowel-welded L-joints for wood furniture. J. Adhes. Sci. Technol..

[139] Renaud A. (2009). Minimalist Z-chair assembly by rotational dowel welding. Eur. J. Wood Prod..

[140] O’Loising C., Oudjene M., Shotton E., Pizzi A., Fanning P. (2012). Mechanical behaviour and 3D stress analysis of multilayered wooden beams made with welded-through wood dowels. Compos. Struct..

[141] O’Loising C., Oudjene M., Ait-Adler H., Fanning P., Pizzi A., Shotton E., Meghlat E.-M. (2012). Experimental study of timber-to-timber composite beam using welded-through wood dowels. Constr. Build. Mater..

[142] Segovia C., Zhou X., Pizzi A. (2013). Wood blockboards for construction by wood welding with pre-oiled dowels. J. Adhes. Sci. Technol..

[143] Mansouri M., Leban J.-M., Pizzi A. (2010). High density panels by wood veneers welding without any adhesives. J. Adhes. Sci. Technol..

[144] Cristescu C., Karlsson O. (2013). Changes in content of furfurals and phenols in self-bonded laminated boards. BioResources.

[145] Je H., Won J. (2020). Natural urushiol as a novel under-water adhesive. Chem. Eng. J..

[146] Bobade S.K., Paluvai N.R., Mohanty S., Nayaka S.K. (2016). Bio-Based Thermosetting Resins for Future Generation: A Review. Polym. -Plast. Technol. Eng..

[147] Sangregorio A., Guigo N., van der Waal J.C., Sbirrazzuoli N. (2019). All ‘green’ composites comprising flax fibres and humins resins. Compos. Sci. Technol..

[148] Sangregorio A., Muralidhara A., Guigo N., Marlair G., Angelici C., Thygesen G., de Jong E., Sbirrazzuoli N. (2020). Humins based resin for wood modification and property improvement. Green Chem..

[149] Hammami N., Jarroux N., Robitzer M., Majdoub M., Habas J.-P. (2016). Optimized Synthesis According to One-Step Process of a Biobased Thermoplastic Polyacetal Derived from Isosorbide. Polymers.

[150] Gu J., Higuchi M., Morita M., Hse C.-Y. (1995). Synthetic conditions and chemical structure of urea-formaldehyde resins. I: Properties of the resins synthesized by three different procedures. Mokuzai Gakkaishi.

[151] Soulard C., Kamoun C., Pizzi A. (1999). Uron and Uron-Urea-formaldehyde resins. J. Appl. Polym. Sci..

[152] Gonçalves C., Pereira J., Almeida M., Carvalho L.H. (2019). Impact of alkaline–acid and strongly acid process on the synthesis of urea–formaldehyde resins and derived composites: A comparison study. Eur. J. Wood Prod..

[153] Lubis M.A.R., Park B.-D. (2020). Enhancing the Performance of Low Molar Ratio Urea–Formaldehyde Resin Adhesives via in-situ Modification with Intercalated Nanoclay. J. Adhes..

[154] Hatam A. (2020). Personal communication.

[155] Dorieh A., Mahmoodi N., Mamaghani M., Pizzi A., Zeydi M.M., Moslemi A. (2019). New insight into the use of latent catalysts for the synthesis of urea formaldehyde adhesives and the mechanical properties of medium density fiberboards bonded with them. Eur. Polym. J..

[156] Dorieh A., Mahmoodi N., Mamaghani M., Pizzi A., Zeydi M.M. (2018). Comparison of the properties of urea-formaldehyde resins by the use of formalin or urea formaldehyde condensates. J. Adhes. Sci. Technol..

[157] Pizzi A., Lipschitz L., Valenzuela J. (1994). Theory and practice of the preparation of low formaldehyde emission UF adhesives for particleboard. Holzforschung.

[158] Dunker A.K., Johns W.E., Rammon R., Farmer B., Johns S.Y. (1986). Slightly Bizarre Protein Chemistry: Urea-Formaldehyde Resin from a Biochemical Perspective. J. Adhes..

[159] Levendis D., Pizzi A., Ferg E.E. (1992). The correlation of strength and formaldehyde emission with the crystalline/amorphous structure of UF resins. Holzforschung.

[160] Ferg E.E., Pizzi A., Levendis D. (1993). A ^13^C NMR analysis method for urea-formaldehyde resin strength and formaldehyde emission. J. Appl. Polym. Sci..

[161] Zanetti M., Pizzi A., Kamoun C. (2003). Upgrading of MUF particleboard adhesives and decrease of melamine content by buffer and additives. Holz Roh-und Werkst..

[162] Zanetti M., Pizzi A. (2003). Upgrading of MUF resins by buffering additives – Part 2: Hexamine sulphate mechanisms and alternate buffers. J. Appl. Polym. Sci..

[163] Despres A., Pizzi A., Delmotte L. (2006). ^13^C NMR investigation of the reaction in water of UF resins with blocked emulsified isocyanates. J. Appl. Polym. Sci..

[164] Wieland S., Pizzi A., Hill S., Grigsby W., Pichelin F. (2006). The reaction in water of UF resins with isocyanates at short curing times: A ^13^C NMR investigation. J. Appl. Polym. Sci..

[165] Wieland S., Pizzi A., Grigsby W., Warnes J., Pichelin F. (2007). Microcristallinity and colloidal peculiarities of UF/isocyanates hybrid resins. J. Appl. Polym. Sci..

[166] Lubis M.A.R., Park B.D., Lee S.M. (2020). Microencapsulation of polymeric isocyanate for the modification of urea-formaldehyde resins. Int. J. Adhes. Adhes..

[167] Martin A.E., Calvin M. (1952). Chemistry of Metal Chelate Compounds.

[168] Lemon P.H.R.B. (1990). An improved sand binder for steel castings. Int. J. Mater. Prod. Technol..

[169] Pizzi A., Stephanou A. (1993). On the chemistry, behaviour and cure acceleration of phenol-formaldehyde resins under very alkaline conditions. J. Appl. Polym. Sci..

[170] Stephanou A., Pizzi A. (1993). Rapid curing lignins-based exterior wood adhesives, Part 2: Acceleration mechanisms and application to panel products. Holzforschung.

[171] Pizzi A., Stephanou A. (1994). Phenol-formaldehyde wood adhesives under very alkaline conditions, Part 2: Acceleration mechanism and applied results. Holzforschung.

[172] Pizzi A., Stephanou A. (1994). Completion of alkaline cure acceleration of phenol-formaldehyde resins: Acceleration by organic anhydrides. J. Appl. Polym. Sci..

[173] Pizzi A., Garcia R., Wang S. (1997). On the networking mechanisms of additives accelerated PF polycondensates. J. Appl. Polym. Sci..

[174] Higuchi M., Tohmura S., Sakata I. (1994). Acceleration of the cure of phenolic resin adhesives 5. Catalytic actions of carbonates and formamide. Mokuzai Gakkaishi.

[175] Conner A.H., Lorenz L.F., Hirth K.C. (2002). Accelerated cure of phenol–formaldehyde resins: Studies with model compounds. J. Appl. Polym. Sci..

[176] Kamo N., Okamura H., Higuchi M., Morita M. (2004). Condensation reactions of phenolic resins V: Cure-acceleration effects of propylene carbonate. J. Wood Sci..

[177] Park B.-D., Riedl B., Hsu E.W., Shields J.A. (1999). Differential scanning calorimetry of phenol–formaldehyde resins cure-accelerated by carbonates. Polymer.

[178] Park B.-D., Riedl B. (2000). ^13^C-NMR study on cure-accelerated phenol–formaldehyde resins with carbonates. J. Appl. Polym. Sci..

[179] Park B.-D., Riedl B., Hsu E.W., Shields J.A. (2001). Application of cure-accelerated phenol-formaldehyde (PF) adhesives for three-layer medium density fiberboard (MDF) manufacture. Wood Sci. Technol..

[180] Lei H., Pizzi A., Despres A., Pasch H., Du G. (2006). Esters acceleration mechanisms in phenol-formaldehyde resin adhesives. J. Appl. Polym. Sci..

[181] Zhao C., Pizzi A., Garnier S. (1999). Fast advancement and hardening acceleration of low condensation alkaline PF resins by esters and copolymerized urea. J. Appl. Polym. Sci..

[182] Zhao C., Pizzi A., Kuhn A., Garnier S. (2000). Fast advancement and hardening acceleration of low condensation alkaline PF resins by esters and copolymerized urea. Part 2: Esters during resin reaction and effect of guanidine salts. J. Appl. Polym. Sci..

[183] Deng S., Du G., Li X., Zhang J., Pizzi A. (2014). Performance and reaction mechanism of zero formaldehyde-emission urea-glyoxal (UG) resin. J. Taiwan Inst. Chem. Eng..

[184] Deng S., Pizzi A., Du G., Zhang J., Zhang J. (2014). Synthesis, structure, and characterization of Glyoxal-Urea-Formaldehyde cocondensed resins. J. Appl. Polym. Sci..

[185] Deng S., Pizzi A., Du G., Lagel M.C., Delmotte L., Abdalla S. (2018). Synthesis, structure characterization and application of melamine-glyoxal adhesive resins. Eur. J. Wood Prod..

[186] Younesi-Kordkheili H., Pizzi A. (2016). Acid Ionic Liquids as a New Hardener in Urea-Glyoxal Adhesive Resins. Polymers.

[187] Xi X., Pizzi A., Amirou S. (2018). Melamine-Glyoxal-Glutaraldehyde Wood Panel Adhesives without Formaldehyde. Polymers.

[188] Novotny E.E., Johnson W.W. (1931). Urea-Furfural Resins and Process of Making Them. U.S. Patent.

[189] Stierli R.F., Newton N.Y. (1949). Urea-Formaldehyde-Furfuryl Alcohol Resins. U.S. Patent.

[190] Lagel M.C. (2016). personal communication.

[191] Xu C., Zhang Y., Yuan Z. (2019). Formaldehyde-Free Phenolic Resins, Downstream Products, Their Synthesis and Use. U.S. patent.

[192] Santiago-Medina F.J., Pizzi A., Abdalla S. (2018). Hydroxymethylfurfural hardening of pine tannin wood adhesives. J. Renew. Mat..

[193] Xi X., Pizzi A. (2020). No-Aldehydes Glucose/Sucrose-Triacetin-Diamine Wood Adhesives for Particleboard. J. Renew. Mat..

[194] Bekhta P., Sedliačik J. (2019). Environmentally-Friendly High-Density Polyethylene-Bonded Plywood Panels. Polymers.

[195] Berthevas P., Santoro G., Wevers R., Gruenbauer H., Pizzi A. Recycled polyurethane foam powder can be used in conjunction with PMDI in particleboards to obtain the required properties while reducing costs. Proceedings of the Ninth European Panel Products Symposium.

[196] Mansouri H.R., Pizzi A. (2007). Recycled polyurethane micronized powders as active extenders of UF and PF wood panel adhesives. Holz Roh-und Werkst..

[197] Ashori A. (2008). Wood–plastic composites as promising green-composites for automotive industries: A review. Bioresour. Technol..

[198] Clemons C. (2002). Wood plastic composites in the United States. The interfacing of two industries. For. Prod. J..

[199] Klyosov A.A. (2007). Wood Plastic Composites.

[200] Schwarzkopf M.J., Burnard M.D., Kutnar A., Muthu S. (2016). Wood-Plastic Composites—Performance and Environmental Impacts. Environmental Impacts of Traditional and Innovative Forest-based Bioproducts.

